# Is it really about freedom of thought?

**DOI:** 10.1192/bjb.2022.9

**Published:** 2022-12

**Authors:** Jack Drescher

**Affiliations:** Columbia University, New York, USA

**Keywords:** Gender incongruence, gender dysphoria, in-patient treatment, adolescents, childhood experience

## Abstract

This opinion piece responds to Marcus Evans's ‘Freedom to Think’ regarding treating adolescents diagnosed with gender dysphoria (DSM)/gender incongruence (ICD). Evans notes not everything is known about GD/GI, particularly its ‘causes’. Although correct, he presents this fact as a rationale for delaying treatment for all children presenting with GD/GI symptoms. However, Marcus does not specify how long such prolonged evaluations should last nor does he have much of an evidence base to support his recommendation. This author believes delaying treatment for GD/GI adolescents who need it for the benefit of children who ‘aren’t really’ transgender is an ethically troubling issue.

I wish to thank *BJPsych Bulletin* for inviting me to respond to Marcus Evans's article about treatment of adolescents with the condition known as gender dysphoria (GD) in DSM-5^1^ and gender incongruence (GI) in ICD-11.^2^

By way of introduction, I am a psychiatrist and psychoanalyst who served on the committees that revised the diagnoses in both those manuals^3,4^ and presently serve as the section editor of the chapter on gender dysphoria in the forthcoming DSM-5 Text Revision (DSM-5-TR; American Psychiatric Press, in press).

Although I am neither a child psychiatrist nor a child analyst, my interest in treatment of this patient population stems from controversies raised in 2008 at the start of the DSM-5 revision process. At that time, charges were made that Kenneth Zucker, who chaired the DSM-5 Workgroup on Sexual and Gender Identity Disorders, was practising ‘conversion therapy’ of transgender minors.^5^ As I have been writing about the harms of conversion therapies since the 1990s,^6,7^ I was surprised to hear this allegation because ‘conversion therapy’ at that time usually referred to attempts to change a homosexual orientation to a heterosexual one and did not include gender identities.

Wishing to learn more about the treatment of minors with GD/GI, my colleague William Byne, MD, and I invited clinicians of different viewpoints to engage in a scholarly discussion on these clinical issues. Five clinical papers followed by six scholarly discussants were first published in a special issue of the *Journal of Homosexuality*^8,9^ and subsequently reissued in an edited book.^10^ I later went on to write about the controversies surrounding the diagnosis and treatment of transgender children and adolescents.^10–12^

## A modest correction: the sky is not falling

Evans begins with a warning that the numbers of people being referred or seeking treatment at gender clinics has risen dramatically in recent years. This is true; and why there has been such an increase in numbers is a question of scientific and clinical curiosity.

So, why has there been an increase in numbers? Neither I nor Evans know the answer to this question and research on it is sparse to non-existent. Theories such as ‘exposure to the internet’ abound, and that may be true in some cases – or not. The answer to the question, at present, is not a scientific fact by any means.

Evans states that ‘Children's sexual orientation and gender identity are formed out of a complex developmental process that involves an interaction between their body, their mind and society at large. Sexual identity and gender identity are developmental processes that evolve as the individual goes through the different life stages’.

As a psychoanalyst, I wouldn't disagree. Where Evans and I part company, however, is that no one – neither he, myself, respected psychoanalytic theorists nor anyone else for that matter – actually knows how those processes unfold. For example, after more than a century of psychoanalytic opining about development of sexual and gender identities, none of these theories have ever been scientifically proven.^13–15^ Yet, Evans cites five references that he calls ‘a growing body of knowledge that connects the development of gender dysphoria with psychological factors’. I am curious as to why Evans is not as troubled as I am by the lack of empirical scientific evidence supporting psychoanalytic theorising about development.

Evans argues that there are parallels between GD/GI and the treatment of anorexia nervosa. As he puts it, ‘We do not just accept/affirm a patient with anorexia when, although she weighs 45 kg, she thinks she is overweight and needs to diet more carefully. Instead, we take it as our duty to try to understand what it is that is driving that belief while persuading her that she needs to eat’. What he says regarding the treatment of anorexia is true, however GD/GI is nothing like anorexia nervosa. The latter is a mental disorder in both DSM and ICD. In WHO's ICD-11, the diagnosis of gender incongruence is no longer a mental disorder^4,12,16^ and historically, the literature on the treatment of the latter has absolutely nothing to do with the treatment of the former.

Evans also cites my friend and colleague James Cantor's (2018) review^17^ of the literature on desistance, referring to about a dozen research studies since the 1970s that show the majority of children with GD/GI grow up to be gay and cisgender (non-transgender). This is also true. Yet, this literature refers to prepubescent children, not adolescents. To be clear, confusing childhood presentations of GD/GI with adolescent ones does not clarify the issues involved.

For example, the presumption that adolescents developing GD/GI without symptoms in childhood means they do not really have that condition is an error. Since 1980, there has been a child diagnosis for prepubescent children and a separate diagnosis for adolescents and adults in both DSM and ICD. It has long been recognised by experts in this area that individuals having either of these two diagnoses, for the most part, constitute different population groups with different developmental trajectories. In fact, GD/GI can appear at almost any age,^18^ including adolescence and adulthood, without any indication of symptoms in childhood. In other words, the existence of adolescents who develop adolescent GD/GI without having had childhood GD/GI is not news at all.

## My opinions

I believe that the so-called gender affirmative model for treating prepubescent children has troubling aspects. For example, there is little empirical evidence that a prepubescent child permitted to socially transition but who later desists can simply and harmlessly transition back to the birth-assigned gender; nor is there any empirically verifiable system of distinguishing persisters from desisters.^19^ I also believe that the Amsterdam clinic's ‘watchful waiting’ approach towards prepubescent children seems best, given the limitations of what we know.^20^ I also do not believe that, as with any major life decision, the decision to gender transition at any age should be done without some modicum of thought and exploration. I also believe that the transgender community's current blanket aversion to what it calls ‘the gatekeeping model’ needs to be rethought. In part this is because, without some modicum of gatekeeping, public trust in clinical decision-making and practices in this area can be and to some extent has been undermined. Nevertheless, I also believe in affirming an adolescent or young adult's gender identity as they go through a thoughtful process of figuring out what they plan to do about it.

In recent years, I am frequently consulted by parents whose prepubescent, adolescent or young adult child has ‘come out’ as transgender. They may regard the child's desire to transition in any the following ways:

- that it just a passing phase their child will outgrow
- they believe their child ‘caught’ the idea from friends
- they believe their child has been ‘brainwashed’ by the internet
- they believe their child has some other psychiatric condition, such as autism or attention deficit disorder, which should preclude any discussion of transition until what they believe to be ‘the underlying condition’ or ‘real reason’ is treated
- they hope their child can be talked out of gender dysphoria
- they think they know their children better than the children know themselves.

In those brief consultations (not treatments), I make efforts to educate parents confused about a complex subject affecting their child, a subject they previously knew nothing about. In some cases, they wished they knew nothing about the subject. Some parents wish to learn something about the issue so they can best assist their child in making the right decision for them. As I've said to some parents, ‘It's not a good thing that your 15-year-old child knows more about this subject than you do’.

Parental concerns are not unimportant and should be taken seriously. In my experience, parents usually want to slow down the transition process. They want to be sure their child is making the right decision and will have no later regrets. They usually want to be sure their child's therapist does not have an agenda to move their child in a direction of which they disapprove. However, more often than not, they are often (but not always) willing to grudgingly accept a child's decision to transition if they trust the child's therapist. This is often a stated reason for why some parents say they have come to me.

Given this responsibility, I try to educate concerned parents with reliable sources of information. The polemics that I believe characterised Evans's article are not the best way to educate parents, the general public, mental health professionals unfamiliar with this patient population, journalists, the courts or policy makers about a complicated issue.

## Concluding remarks

In recent years I have been invited to speak in the USA and in other countries about the controversies surrounding transgender children and adolescents. To my knowledge, I have never been silenced for any of my views, not all of which are accepted by activist members of the transgender community. And his dramatic claims notwithstanding, given that both *BJPsych Bulletin* and the UK's judicial system have given Evans prominent forums for expressing his views, apparently neither has he.

Evans correctly points out that we do not know everything about GD/GI, particularly its ‘causes’. However, he presents this fact as a rationale for delaying treatment for children with GD/GI who may need it. By this reasoning, if I may be allowed a medical analogy of my own, no one should treat idiopathic hypertension because we do not know its causes.

Evans writes: ‘Whatever decisions are made regarding medical treatment, a thorough psychotherapeutic and psychiatric assessment is essential to enable us to help these vulnerable young people, their families and their clinical teams make informed decisions’. I completely agree with this statement. However, what he does not specify is how long such an evaluation should last. A month? Three months? A year? Longer? A longer approach may benefit children who might *not* grow up to be transgender. However, delaying treatment for all children inevitably comes at the expense of those who will remain gender dysphoric. In my opinion, clinicians delaying treatment for GD/GI adolescents who need it and may benefit from it in order to ‘protect’ those children who ‘aren't really’ transgender is an ethically troubling issue. In other words, ‘first, do no harm’ is a sword that can cut two ways.

## About the author

**Jack Drescher**, MD, is Clinical Professor of Psychiatry in the Department of Psychiatry at Columbia University, New York, NY, USA.

## References

[1] American Psychiatric Association. Diagnostic and Statistical Manual of Mental Disorders (5th edn) (DSM-5). American Psychiatric Press, 2013.

[2] World Health Organization. ICD-11 for Mortality and Morbidity Statistics (ICD-11 MMS) (version: 05/2021). WHO, 2021 (https://icd.who.int/browse11/l-m/en).

[3] Zucker KJ, Cohen-Kettenis PT, Drescher J, Meyer-Bahlburg HFL, Pfäfflin F, Womack WM. Memo outlining evidence for change for gender identity disorder in the DSM-5. Arch Sex Behav 2013; 42: 901–14. 23868018 10.1007/s10508-013-0139-4

[4] Reed GM, Drescher J, Krueger RB, Atalla E, Cochran SD, First MB, Revising the ICD-10 Mental and Behavioural Disorders classification of sexuality and gender identity based on current scientific evidence, best clinical practices, and human rights considerations. World Psychiatry 2016; 15: 205–21. 27717275 10.1002/wps.20354 PMC5032510

[5] Drescher J. Queer diagnoses: parallels and contrasts in the history of homosexuality, gender variance, and the *Diagnostic and Statistical Manual* (DSM). Arch Sex Behav 2010; 39: 427–60. 19838785 10.1007/s10508-009-9531-5

[6] Drescher J. I'm your handyman: a history of reparative therapies. J Homosex 1998; 36: 19–42. 10.1300/J082v36n01_02 9670099

[7] Drescher J, Schwartz A, Casoy F, McIntosh CA, Hurley B, Ashley K, The growing regulation of conversion therapy. J Med Regul 2016; 102: 7–12. 27754500 PMC5040471

[8] Drescher J, Byne W. Introduction to the treatment of gender dysphoric/gender variant (GD/GV) children and adolescents. J Homosex 2012; 59: 295–300. 22455321 10.1080/00918369.2012.653299

[9] Drescher J, Byne W. Gender dysphoric/gender variant (GD/GV) children and adolescents: summarizing what we know and what we have yet to learn. J Homosex 2012; 59: 501–10. 22455333 10.1080/00918369.2012.653317

[10] Drescher J, Byne W (eds). Treating Transgender Children and Adolescents: An Interdisciplinary Discussion. Routledge, 2013.

[11] Drescher J. Controversies in gender diagnoses. J LGBT Health 2014; 1: 9–15. 10.1089/lgbt.2013.1500 26789504

[12] Drescher J, Cohen-Kettenis PT, Reed GM. Gender incongruence of childhood in the ICD-11: controversies, proposal, and rationale. Lancet Psychiatry 2016; 3: 297–304. 26946394 10.1016/S2215-0366(15)00586-6

[13] Drescher J. Psychoanalytic Therapy and the Gay Man. Routledge, 2001. 10.1023/a:1001968909523 10874429

[14] Drescher J. Causes and becauses: on etiological theories of homosexuality. Ann Psychoanal 2002; 30: 57–68.

[15] Drescher J. A history of homosexuality and organized psychoanalysis. J Am Acad Psychoanal Dyn Psychiatry 2008; 36: 443–60. 18834283 10.1521/jaap.2008.36.3.443

[16] Drescher J, Cohen-Kettenis PT, Winter S. Minding the body: situating gender diagnoses in the ICD-11. Int Rev Psychiatry 2012; 24: 568–77. 23244612 10.3109/09540261.2012.741575

[17] Cantor J. American Academy of Pediatrics policy and trans-kids: fact-checking. Sexol Today! 2018; 17 Oct (http://www.sexologytoday.org/2018/10/american-academy-of-pediatrics-policy.html).

[18] Nieder TO, Herff M, Cerwenka S, Preuss WF, Cohen-Kettenis PT, De Cuypere G, Age of onset and sexual orientation in transsexual males and females. J Sex Med 2011; 8: 783–91. 21143416 10.1111/j.1743-6109.2010.02142.x

[19] Drescher J, Pula J. Ethical issues raised by the treatment of gender variant prepubescent children. Hastings Cent Rep 2014; 44(suppl 4): S17–22. 25231780 10.1002/hast.365

[20] de Vries AL, Cohen-Kettenis PT. Clinical management of gender dysphoria in children and adolescents: the Dutch approach. J Homosex 2012; 59: 301–20. 22455322 10.1080/00918369.2012.653300

